# A snake venom-analog peptide that inhibits SARS-CoV-2 and papain-like protease displays antithrombotic activity in mice arterial thrombosis model, without interfering with bleeding time

**DOI:** 10.1186/s12959-022-00436-5

**Published:** 2023-01-02

**Authors:** Ruben Siedlarczyk Nogueira, Bruno Ramos Salu, Vinícius Goulart Nardelli, Camila Ramalho Bonturi, Marina Rodrigues Pereira, Francisco Humberto de Abreu Maffei, Eduardo Maffud Cilli, Maria Luiza Vilela Oliva

**Affiliations:** 1 Department of Biochemistry, Universidade Federal de São Paulo (UNIFESP), SP 04044- 020 São Paulo, Brazil; 2 Department of Biochemistry and Organic Chemistry, Institute of Chemistry, Universidade Estadual Paulista (UNESP), SP 14800-060 São Paulo, Araraquara, Brazil; 3 Department of Surgery and Orthopedics, Universidade Estadual Paulista (UNESP), 18618-687 São Paulo, Botucatu, SP Brazil

**Keywords:** Bleeding, COVID-19, Cysteine-protease, Papain-like protease, Platelets, SARS-CoV-2, Thrombosis

## Abstract

**Background:**

(p-BthTX-I)_2_ K, a dimeric analog peptide derived from the C-terminal region of phospholipase A2-like bothropstoxin-I (p-BthTX-I), is resistant to plasma proteolysis and inhibits severe acute respiratory syndrome coronavirus 2 (SARS-CoV-2) strains with weak cytotoxic effects. Complications of SARS-CoV-2 infection include vascular problems and increased risk of thrombosis; therefore, studies to identify new drugs for treating SARS-CoV-2 infections that also inhibit thrombosis and minimize the risk of bleeding are required.

**Objectives:**

To determine whether (p-BthTX-I)_2_ K affects the hemostatic system.

**Methods:**

Platelet aggregation was induced by collagen, arachidonic acid, and adenosine diphosphate (ADP) in the Chronolog Lumi-aggregometer. The coagulation activity was evaluated by determining activated partial thromboplastin clotting time (aPTT) and prothrombin time (PT) with (p-BthTX-I)_2_ K (5.0–434.5 µg) or 0.9% NaCl. Arterial thrombosis was induced with a 540 nm laser and 3.5–20 mg kg^− 1^ Rose Bengal in the carotid artery of male C57BL/6J mice using (p-BthTX-I)_2_ K. Bleeding time was determined in mouse tails immersed in saline at 37 °C after (p-BthTX-I)_2_ K (4.0 mg/kg and 8.0 mg/kg) or saline administration.

**Results:**

(p-BthTX-I)_2_ K prolonged the aPTT and PT by blocking kallikrein and FXa-like activities. Moreover, (p-BthTX-I)_2_ K inhibited ADP-, collagen-, and arachidonic acid-induced platelet aggregation in a dose-dependent manner. Further, low concentrations of (p-BthTX-I)_2_ K extended the time to artery occlusion by the formed thrombus. However, (p-BthTX-I)_2_ K did not prolong the bleeding time in the mouse model of arterial thrombosis.

**Conclusion:**

These results demonstrate the antithrombotic activity of the peptide (p-BthTX-I)_2_ K possibly by kallikrein inhibition, suggesting its strong biotechnological potential.

## Highlights

• Venom-analog peptide, (p-BthTX-I)_2_K, inhibits SARS-CoV-2 infection and papain-like protease.

• Bothropstoxin-I-derived peptide analog shows antithrombotic action.

• (p-BthTX-I)_2_K prolonged aPTT and PT by blocking kallikrein and factor Xa.

• Venom-analog peptide, (p-BthTX-I)_2_K, shows antithrombotic action without prolonging bleeding time.

## Introduction

Patients infected by severe acute respiratory syndrome coronavirus 2 (SARS-CoV-2) are at risk for vascular complications [1]. Those who develop the disease show alterations in one or more components of Virchow’s triad. Endothelial injury, blood stasis, and decreased blood flow velocity within the vessels represent imbalances in Virchow’s triad, and a greater propensity for clot formation arises [2]. SARS-CoV-2 infection increases the risk of thrombosis because it promotes hematological alterations and hypercoagulability. Clotting abnormalities, such as longer prothrombin time (PT) and activated partial thromboplastin clotting time (aPTT) are evident shortly after patient hospitalization, and fibrinogen concentration and antithrombin activity decrease over time in some patients [3]. Elevations of D-dimer levels are indicators of adverse outcomes for COVID-19 patients, given that D-dimer is a product of fibrin degradation and may be elevated due to concomitant activation of fibrinolysis during thrombus formation [4]. Although there is evidence of an increased risk of thrombosis in COVID-19 patients, complete anticoagulation treatment for all patients is questionable and has not been recommended by the international community [5]. Therefore, research to identify new drugs against COVID-19 with the antithrombotic effect but that minimize bleeding is essential. To study new antimicrobial molecules derived from snake venom toxins, Santos-Filho et al. [6] synthesized a monomeric peptide p-BthTX-I (sequence: KKYRYHLKPFCKK) from the C-terminal region of the phospholipase A2-like BthTX-I [7]. The peptide p-BthTX-I has a cysteine residue in its sequence. The recognition of the antimicrobial activity of p-BthTX-I was realized by synthesizing and studying a homodimeric version of p-BthTX-I, (p-BthTX-I)_2_, which is linked through cysteine-cysteine residues by oxidation. In this study, the peptide’s dimeric form, (p-BthTX-I)_2_, showed the highest cytotoxicity against bacteria, indicating that the cysteine residue and dimerization are essential for its cytotoxic activity. In contrast, it does not promote toxic effects in eukaryotic cells [6].

The development of antimicrobial peptides for clinical use has been limited because peptides are proteolytically degraded in the circulatory system. Santos-Filho et al. [8] aimed to characterize the degradation profile of (p-BthTX-I)_2_ and reported that (p-BthTX-I)_2_ was degraded after serum incubation; however, the primary degradation product was a peptide with lost four lysine residues in the C-terminus region ((des-Lys^12^, Lys^13^-(p-BthTX-I)_2_). The peptides ((des-Lys^12^, Lys^13^-(p-BthTX-I)_2,_ and (p-BthTX-I)_2_ showed antimicrobial activity against a range of bacterial strains, including multidrug-resistant bacteria. Regarding the mechanism of action (MoA) of these molecules, Santos-Filho et al. [9] showed that (p-BthTX-I)_2_ acts differently against gram-negative (*Escherichia coli*) and gram-positive (*Staphylococcus aureus*) bacteria.

To study the structure-function relationship of this class of molecules, Santos-Filho et al. [10] synthesized several (p-BthTX-I)_2_ analogs, including the dimer Lys-linker peptide (des-Cys^11^, Lys^12^, Lys^13^-(p-BthTX-I))_2_ K [(KKYRYHLKPF)_2_ K] named as (p-BthTX-I)_2_ K. Their synthesis method avoided cysteine oxidation, which decreased one step in the original synthesis method and resulted in a smaller and more stable peptide. Interestingly, the antimicrobial activity of this peptide (p-BthTX-I)_2_ K was superior to or like that of (p-BthTX-I)_2_, with no increased hemolytic activity. (p-BthTX-I)_2_ K and its analogs showed inhibitory activity against SARS-CoV-2 with a low cytotoxic effect. In addition, (p-BthTX-I)_2_ K inhibits papain-like protease (PL^pro^) in the low micromolar range against SARS-CoV-2; and weak inhibitory activity against M^pro^, showing selective target being a promising prototype to explore for new drugs against SARS-CoV-2 infection [11].

As mentioned previously, arterial and venous thrombosis are the final trigger for complications and mortality in COVID-19 patients. Therefore, we investigated the effect of (p-BthTX-I)_2_ K on coagulation parameters and platelet function in vitro and the antithrombotic effect in *vivo* using a mice model of arterial thrombosis.

## Materials and methods

### Human blood clotting parameters

Blood was collected from healthy and unmedicated volunteers in polypropylene tubes containing 3.8% sodium citrate (1/10, w/v). The blood sample was centrifuged at 350 x*g* for 15 min at 25 °C, to obtain platelet-poor plasma (PPP), and PPP was stored at ^−^80 °C until use. The experimental protocols were conducted according to the guidelines of the ethics committee of the Universidade Federal de São Paulo, São Paulo, Brazil.

### Activated partial thromboplastin time and prothrombin time determination

aPTT and PT assays were performed as described by Brito et al. [12] with minor alterations. To determine aPTT, we incubated 50 µL (16.3 up to 217.4 µg) of (p-BthTX-I)_2_ K or 0.15 M NaCl (control) with 50 µL of PPP and 50 µL of aPTT reagent for 2 min followed by the addition of 50 µL of 0.025 M calcium chloride. The clotting time was measured in seconds (sec). PT was assayed by incubating 50 µL of (p-BthTX-I)_2_ K (108.7–434.8 µg) with 50 µL of PPP for 60 s, followed by the addition of 100 µL of Thromborel S reagent (Siemens Healthineers, Marburg, Germany). Assays were performed in duplicates and the results were expressed in seconds.

### Evaluation of (p-BthTX-I)_2_ in plasma-like activities of kallikrein and factor Xa

Preincubation of 20 µL of human plasma was performed with increasing concentrations of (p-BthTX-I)_2_ K (5.0–80 µg) or 0.15 M NaCl solution and 10 µL of aPTT activator reagent (actin activated cephaloplastin, Dade Behring, Marburg, Germany) for 10 min at 37 ºC. To determine the hydrolytic activity, 20 µL of this plasma was mixed with 0.7 mM H-D-Pro-Phe-Arg-pNan (substrate for kallikrein-like enzymes) or 2 mM CH_3_OCO-D-CHA-Gly-Arg-pNa-AcOH (FXa-like enzymes) (Behring, Marburg, Germany) in 0.02 M Tris-HCl, 140 mM NaCl, 5 mM CaCl_2_, 0.1% BSA (pH 7.4) (FXa) or 0.05 M Tris-HCl, 120 mM NaCl, 0.1% BSA (PKa) and subsequently activated with 10 µL of 0.025 M calcium chloride. Substrate hydrolysis was monitored for 60 min by photometric reading at 405 nm using the SpectraMax Plus 384 Microplate Reader (Molecular Devices, San Jose, CA, USA).

### Measurement of nitric oxide

Nitric oxide (NO) is an important molecule that has been extensively investigated due to its wide-ranging physiological and biological involvement. In biological fluids such as plasma, NO is rapidly converted to oxidation products (NO_2_^−^ and NO_3_^−^), which emit photons into the ground state and these photons can be measured by a photomultiplier tube (PMT). The proportion of NO in the reaction cell is equivalent to the emitted light detected by the PMT [13]. To test NO production, we incubated pooled plasma obtained from healthy donors at COLSAN with increasing concentrations of (p-BthTX-I)_2_ K (5.0 up to 80 µg) for 2 h, froze the solutions, and stored them at -20 °C until use. For deproteinization, ethanol was added to the plasma sample (1:2 ratio) and incubated for 30 min at 4 °C. Next, samples were centrifuged for 5 min at 17,000 x*g* at 4 °C. Total protein was quantified in the clear supernatant, and 100 µL aliquots were used to measure total NO oxidation products with a nitric oxide analyzer (NOA, model 280, Sievers Instruments, Boulder, CO, USA) [14]. NO concentrations were expressed as micromoles (µM).

### Human platelet aggregation

Platelet aggregation was measured using platelet-rich plasma (PRP) obtained from pooled venous blood of healthy donors collected in conical plastic tubes containing 3.8% trisodium citrate (1:10, v/v). PRP was obtained by centrifugation at 350 x*g* for 12 min at 25° C. Concentration of 2.5 × 10^8^ platelets/mL was maintained with HEPES Tyrode buffer (pH 7.4) (137 mM NaCl, 2.9 mM KCl,12.0 mM Na_2_HPO_4_, 1.0 mM MgCl_2_, 5 mM HEPES, 5 mM C_6_H_12_O_6_, and 1 mM CaCl_2_) using a hematological cell counter (Sysmex KX-21 N™, Sysmex, Kobe, Hyogo Japan) [15]. PRP aggregation was performed using the Chrono-Log 490 aggregometer (Chrono-Log, Havertown, PA) with continuous shaking at 37 °C and 160 x*g*. The agonists used were adenosine diphosphate (ADP) (5 µM) (Chrono-Log, Havertown, PA, USA), collagen (2 µg/mL) (Chrono-Log), and arachidonic acid (1.0 mM) (Chrono-Log). HEPES buffer was used as an experimental control, and the final volume of the reaction was 500 mL. Aggregation was monitored for 5 min and 30 s. The degree of platelet aggregation was defined as the percentage change in light transmittance from PRP (0% light transmission) to PPP (100% light transmission).

To test the effect of the peptide (p-BthTX-I)_2_ K, we pre-incubated platelets with increasing concentrations of (p-BthTX-I)_2_ K (7.82–608.7 µg) for 5 min at 37 °C under agitation before adding the agonists. Control of platelet aggregation was performed at the beginning and end of each experiment to confirm platelet viability using 0.1 IU Thrombin (Chrono-Log).

### *Ex vivo* and *in vivo* experimental models

In order to study the experimental mouse model of arterial thrombosis, we obtained C57BL/6 male adult mice that were 7–8 weeks old and that weighed 20–25 g from the Instituto de Farmacologia e Biologia Molecular Professor Dr. Ribeiro do Vale at Universidade Federal de São Paulo (INFAR-UNIFESP). Animal experimentation protocols were approved by the Committee on Ethics in the Use of Animals (CEUA) process number 2019-7922240819, and all protocols followed the Animal Research: Reporting of *In Vivo* Experiments (ARRIVE) guidelines.

In all experiments, mice were intraperitoneally anesthetized before experiments with ketamine (100 mg/kg), xylazine (20 mg/kg), and morphine (5 mg/kg). The experiments were initiated 10 min after administering anesthesia. During the procedures, the animals were kept warm in a thermal blanket. At the end of the experiment, the animals were euthanized with an excessive dose of ketamine (300 mg/kg) and xylazine (30 mg/kg).

### Arterial thrombosis induction

Arterial thrombosis was induced by photochemical damage to the endothelium as described [16], with minor modifications as described by Salu et al. [17]. The left carotid artery was isolated, and a *mid-*cervical incision was made with the help of a microscope. Next, (p-BthTX-I)_2_ K (3.75–20.0 mg/kg) or NaCl (0.15 M) was administered intravenously via retro-orbital injection, and after 10 min, Rose Bengal (50 mg/kg) (3,4,5,6 tetrachloro-2,4,5,7-tetraiodofluorescein, Sigma Aldrich, Saint Louis, MO, USA) was administered. An ultrasonic flowmeter (model MA 0.5 PSB; Transonic System, Ithaca, NY) was then placed around the artery, and a 1.5 mW, 540 nm laser was used to irradiate the carotid artery from a distance of 6 cm. The blood flow rate was monitored continuously for 5 min until it stabilized at 0.05 mL/min or lower.

### Bleeding time

The animals were anesthetized with ketamine (100 mg/kg) and xylazine (20 mg/kg), and 90 min after administering (p-BthTX-I)_2_ K (4.0 mg/kg and 8.0 mg/kg) or saline solution (control), a longitudinal incision of 2 mm was made at the end of mice tail. The incised tail was immersed in saline solution (0.15 M NaCl) at 37 °C. The time until bleeding ceased for more than 30 s was measured; for longer bleeding times, a value of 10 min was adopted [18].

### Data analysis

Graphs represent mean ± S. D and were built in GraphPad Prism 8 (GraphPad Software, San Diego, CA). Statistical analysis was performed using *Jamovi* (The Jamovi project – 2021) version 1.6. Distribution tests were performed using the Shapiro-Wilk test, and group comparisons for non-parametric distributions (bleeding time, PT, aPTT) were performed using the Mann-Whitney test. For samples with a normal distribution, an independent Student’s t-test was performed. In the arterial thrombosis experiment, groups were compared using a two-way analysis of variance test (ANOVA) followed by a Tukey-Kramer post-test.

## Results

### (p-BthTX-I)_2_K interacts with coagulation enzymes and enhances aPTT and PT

**Fig. 1:**
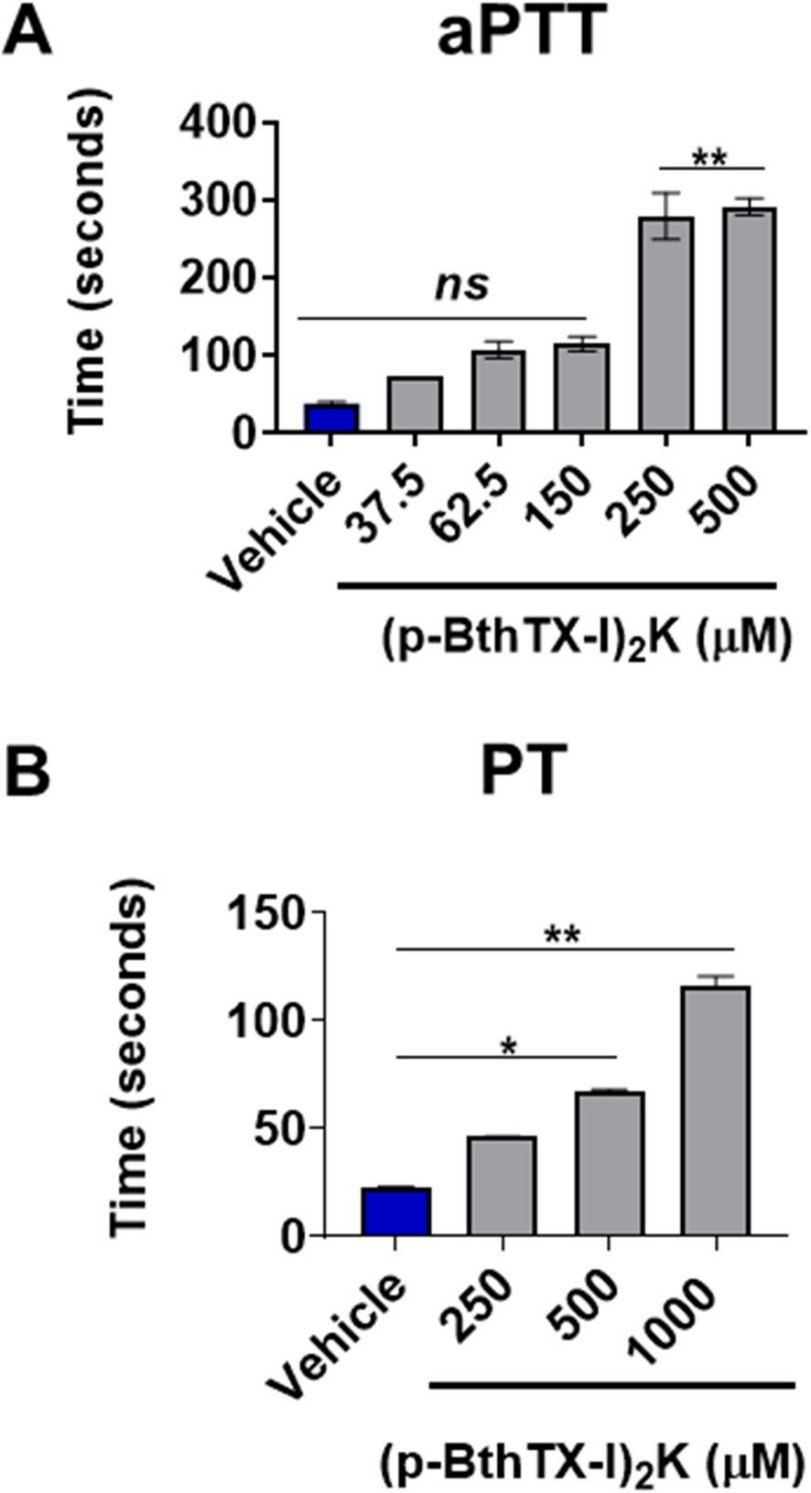
Effects of the peptide (p-BthTX-I)_2_K in the aPTT, and PT parameters. aPTT or PT was determined using a plasma pool of healthy human volunteers (*n* = 6). The plasma was tested with saline (vehicle) or peptide. Data are expressed as mean ± standard deviation, and *p˂0.05, **p˂0.01 considering the comparison of each peptide amount with NaCl 0.15 M in the Mann-Whitney test

**Fig. 2:**
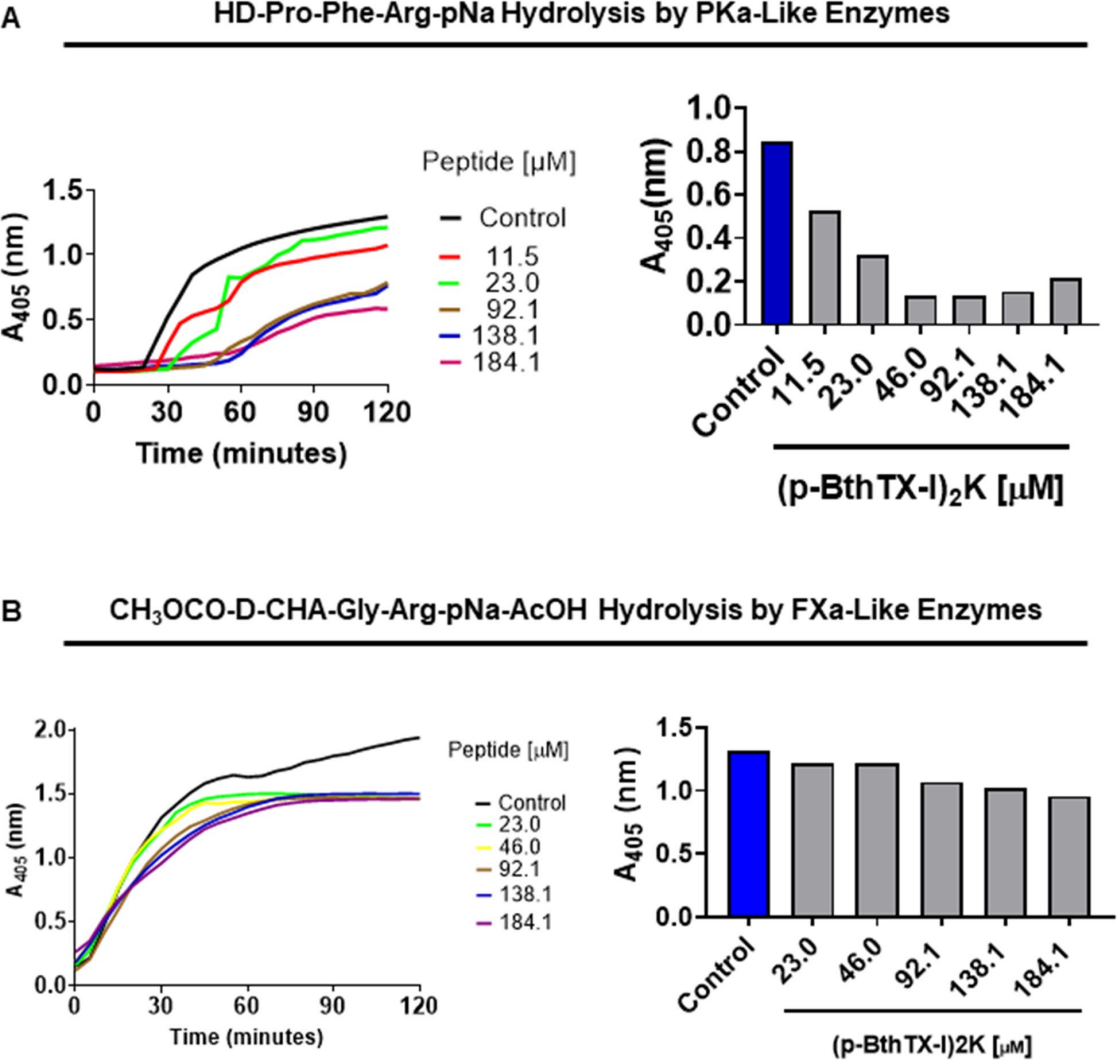
Influence of (p-BthTX-I)_2_ K on the enzymatic activities of plasma serine proteases. Plasma enzymes were pre-activated as described in the methods and pre-incubated (10 min, at 37 °C) in presence of increasing amounts of (p-BthTX-I)_2_ K) and substrate hydrolysis was monitored for about 60 or 120 min. Residual activity was measured using (**A**) 2 mM HD-Pro-Phe-Arg-pNan or (**B**) 5 mM CH_3_OCO-D-CHA-Gly-Arg-pNa-AcOH, substrates for PKa and FXa, respectively. Bars graphs represent the endpoint at 60 min compared to 0.15 M NaCl. The experiment was repeated three times

(p-BthTX-I)_2_ K prolonged both aPTT and PT. From the concentration of 108.7 µg onwards, we observed an increase in both aPTT and PT, especially in the aPTT, with a 10-fold rise. In PT assay, this pattern was observed since 1-fold enhancement was observed (Fig. 1 A, B). The significant increase in the aPTT was confirmed by inhibiting the intrinsic (Fig. 2 A) and extrinsic (Fig. 2B) pathway enzyme activities since a decrease in hydrolysis by the enzymes of the synthetic substrates HD-Pro-Phe-Arg-pNa and CH_3_OCO-D-CHA-Gly-Arg-pNa-AcOH presence of the peptide was observed.

### (p-BthTX-I)_2_K suppress with nitric oxide release *in vitro*

**Fig. 3:**
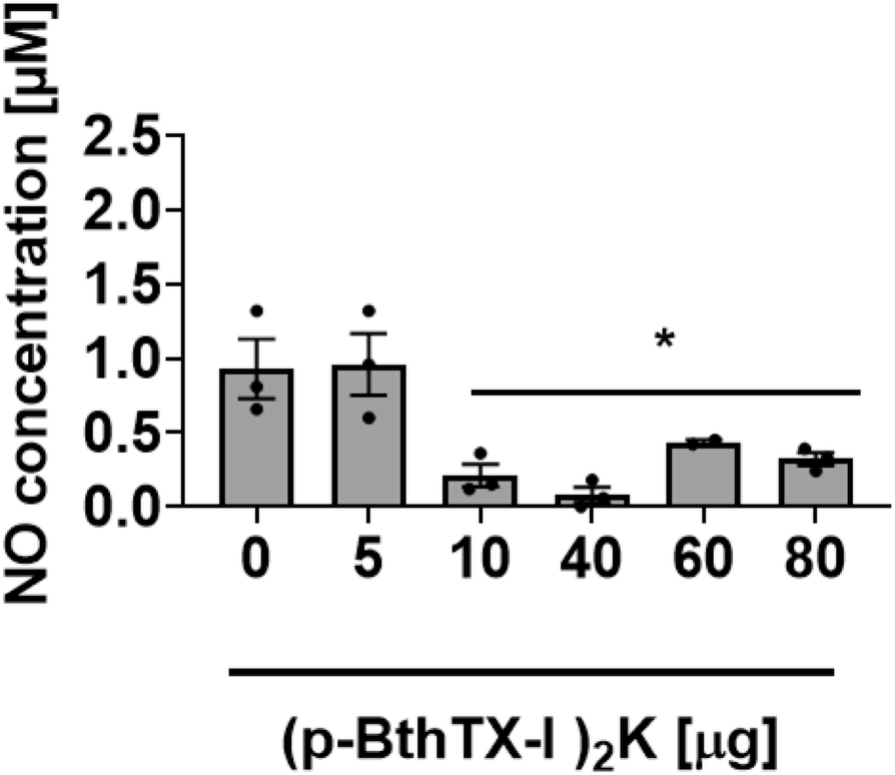
Influence of (p-BthTX-I)_2_ K on NO formation in plasma. The level of NO production was determined in human plasma incubates or not (control), for 2 h at room temperature with an increased amount of peptide. Data are expressed as mean ± S.D, *p* < 0.05, in Mann-Whitney test

As shown in Fig. 3, NO levels lowered in the(p-BthTX-I)_2_ K incubated plasma within two hours at 25 °C with peptides from 10 µg up to 80 µg. At (p-BthTX-I)_2_ K amounts of 10 µg, 40 µg, 60 µg, and 80 µg, an intense decrease in NO concentration was observed. These findings corroborate our previous results, indicating that (p-BthTX-I)_2_ K by inhibiting plasma kallikrein may interfere with NO synthesis.

### (p-BthTX-I)_2_K impairs *in vitro *platelet aggregation

**Fig. 4:**
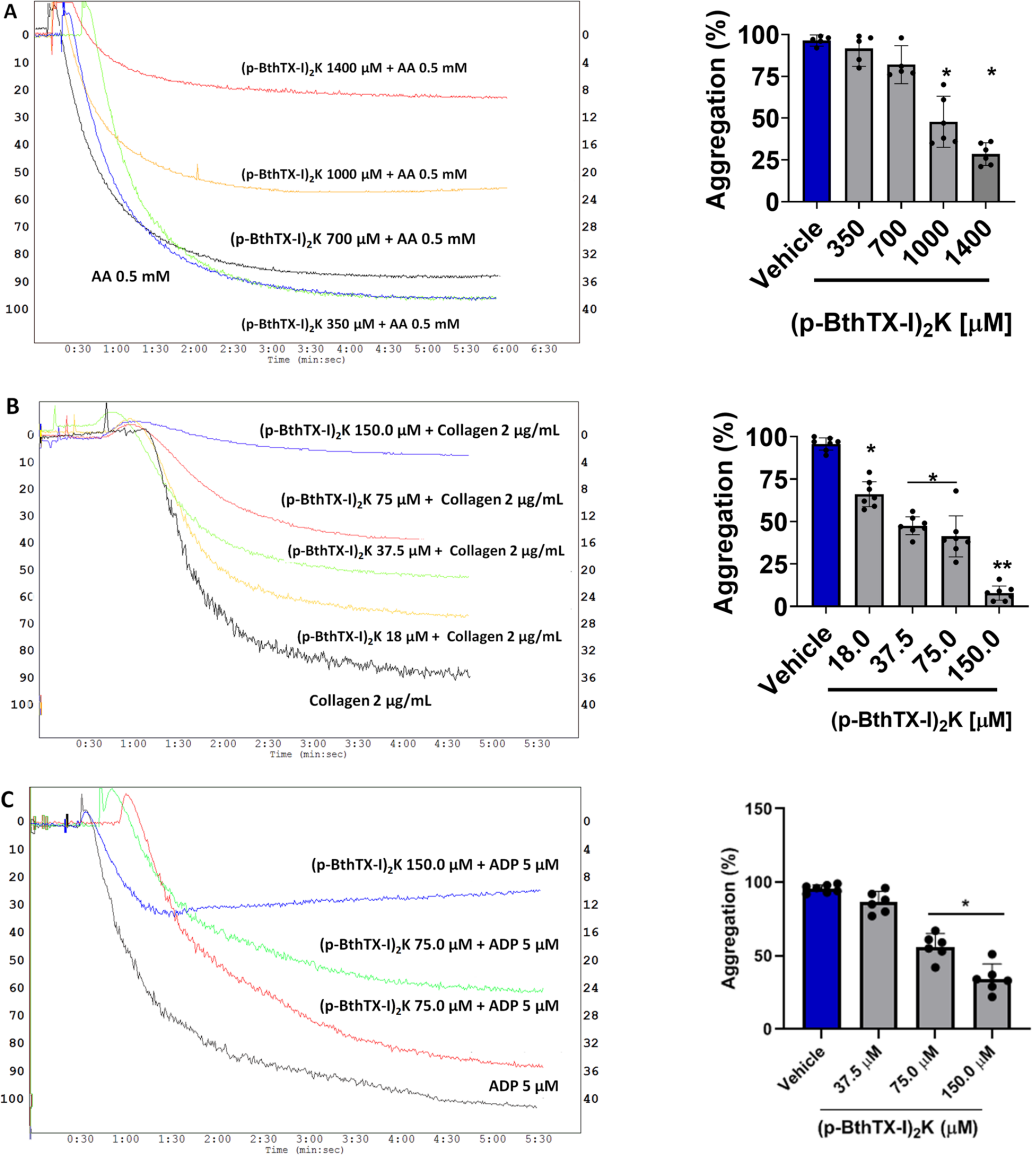
Effect of (p-BthTX-I)_2_ K on platelet aggregation. The compound provoked a decline in platelet aggregation with arachidonic acid, collagen, and ADP (**A-C**) after incubation with 434.8 and 608.7 µg (arachidonic acid) or 32.6 and 65.2 µg (collagen and ADP). Statistical analysis by comparing the groups with Student t-test with *p* < 0.001. Curves refer to platelet aggregation assays demonstrating the dose-dependent effect of the peptide on the aggregation of human platelets

(p-BthTX-I)_2_ K was able to prevent platelet aggregation induced by three important agonists: ADP, arachidonic acid, and collagen (Fig. 4 A-C). The peptide effectively inhibited ADP-mediated platelet aggregation in a dose-dependent manner (Fig. 4-C). Using collagen as a platelet agonist (Fig. 4-B), it was verified that the peptide was also very effective when used at amounts ranging from 7.82 to 65.2 µg. Arachidonic acid-induced platelet aggregation was also affected by (p-BthTX-I)_2_ K (Fig. 4-A).

### (p-BthTX-I)_2_ K) impairs arterial thrombus formation in mice

**Fig. 5:**
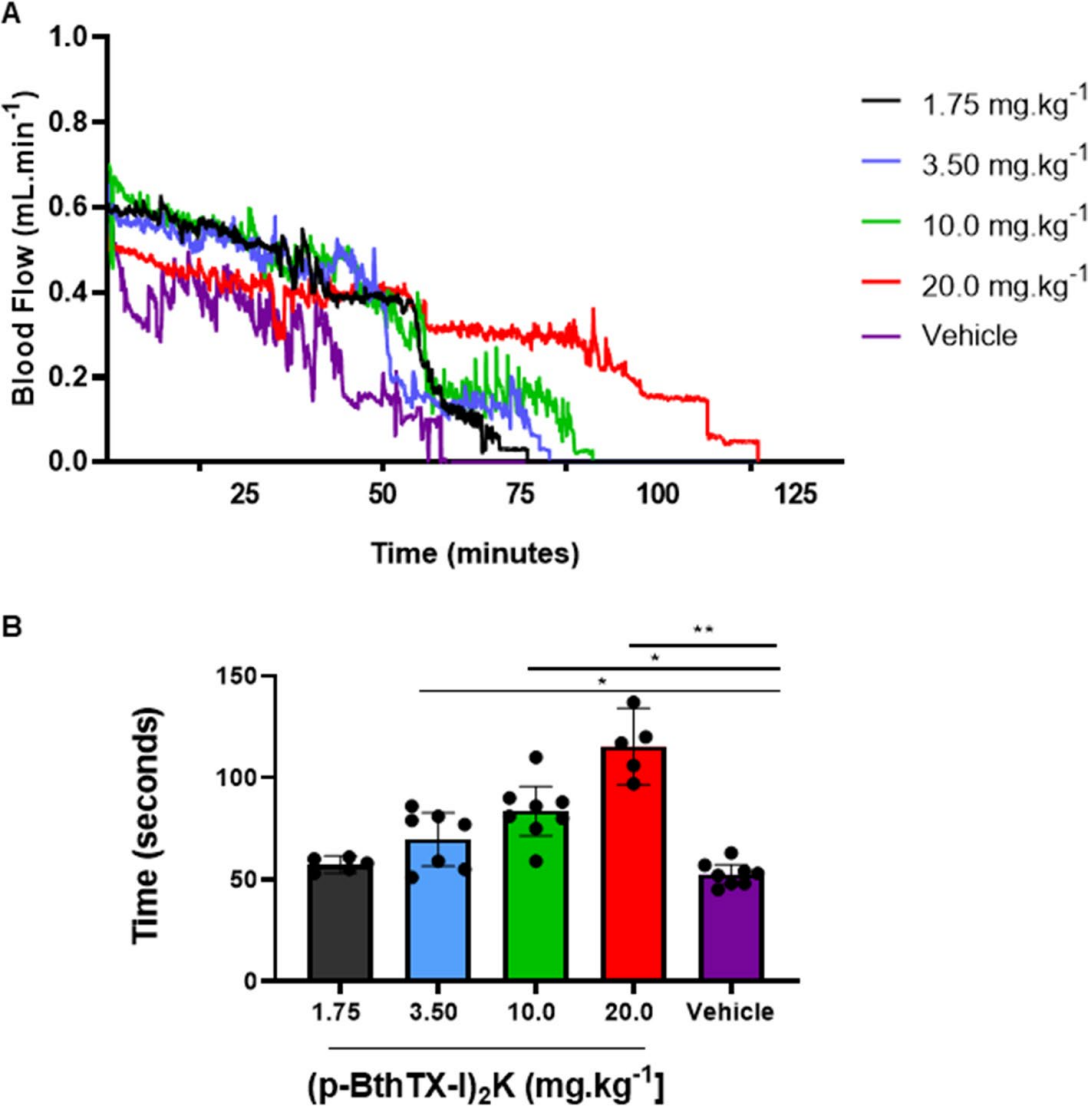
Effect of (p-BthTX-I)_2_ K on mice photochemical arterial thrombosis model. **A **Representative graph of the blood flow in the carotid artery of the groups of animals submitted to induction of arterial thrombosis by the photosensitizing method. In purple, the 0.9% NaCl group (*n* = 8). In red (*n* = 5) (p-BthTX-I)_2_ K 20.0 mg.kg^− 1^ group. Black, blue and green lines represent intermediate amounts of the peptide. **B **Box plot representing the carotid artery occlusion time of mice that received (p-BthTX-I)_2_ K at amounts from 1.75 mg/kg up to and 20.0 mg/kg (*n* = 5 animals each), and Vehicle (0.9% NaCl). Statistical analysis was performed using two-way ANOVA and multiple comparisons, with *p* < 0.01 being significant

Owing to evidence of thrombotic complications in a patient with COVID-19, we tested the possible antithrombotic properties of (p-BthTX-I)_2_ K in a mouse model of arterial thrombosis by comparing it with its vehicle (0.9% NaCl). Figure 5(A) and **(**B**)** show the ability of the peptide to prolong the time to arterial thrombus formation in a statistically significant way and in a dose-dependent manner (two-way ANOVA with a significance of *p* < 0.01).

### (p-BthTX-I)_2_ K) impaired blood flow blockage without causing bleeding

**Fig. 6:**
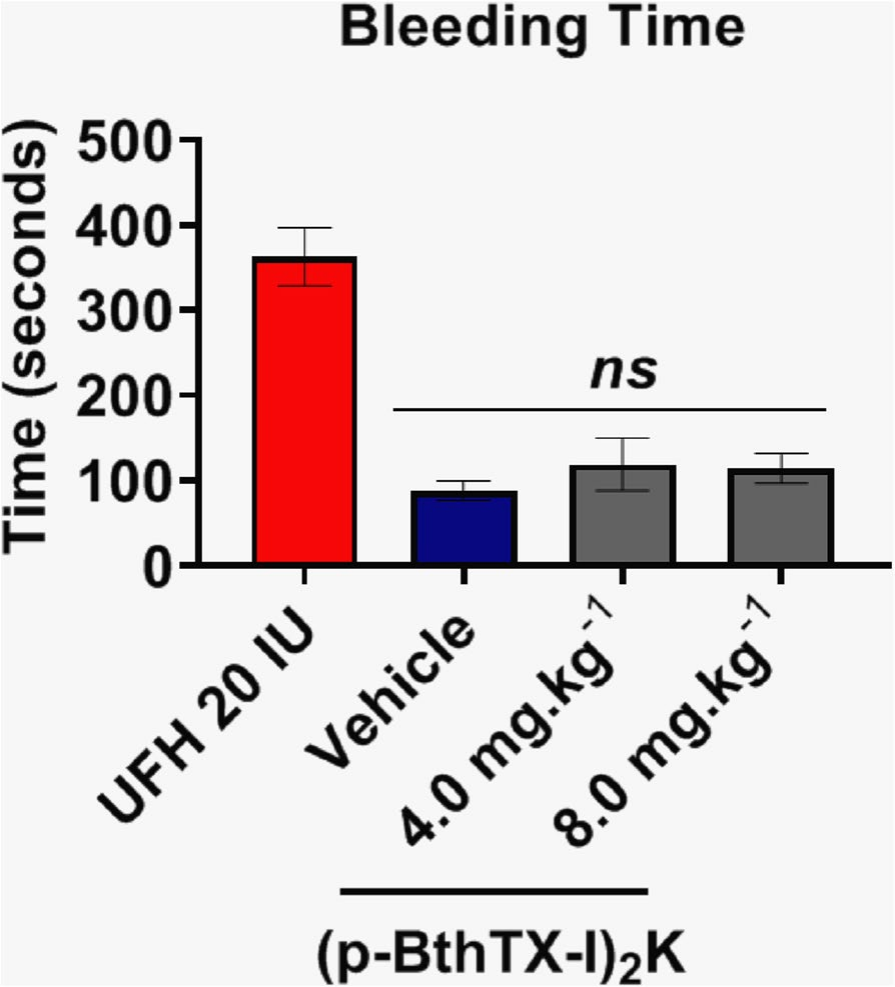
Bleeding time. Mice were treated with (p-BthTX-I)_2_ K or heparin. Transection of the tail was performed as described and the bleeding time was recorded until bleeding ceased more than 30 s. There is no significant difference between the peptide-treated group and saline determined using the Mann-Whitney test

The bleeding time in (p-BthTX-I)_2_ K-treated animals (4.0 mg/kg and 8.0 mg/kg) did not change when compared to the saline-treated group and was significantly lower than that in mice treated with 20 IU of unfractionated heparin (Fig. 6). There was no significant difference when we compared both groups using the Mann-Whitney test.

## Discussion

Thrombus formation is a consequence of an imbalance in the hemostatic system. Its normal function is to keep the blood fluid circulating in the vessels and to act when endothelium lesions occur [19]. Several proteases and protease inhibitors are present in the circulatory system, such as proteases from the inside and released by white and red blood cells, platelets, and endothelium that are all involved in hemostatic mechanisms [3].

Many researchers have reported that systemic pathological events (excessive immune response, hyper-inflammation, and release of proinflammatory cytokines and chemokines) in COVID-19 infection trigger macro and microvascular thrombosis [20–22]. Additionally, the recruitment and activation of leukocytes and platelets result in the progression of coagulopathy via intravascular generation of thrombin. This event further activates endothelial cells, platelets, and leukocytes in a continuous feedback loop, generating thrombin and increasing the clinical manifestations of viral coagulopathy, including venous and arterial thrombosis, thromboembolism, and microvascular thrombosis.

Complement activation also plays a prothrombotic role by recruiting leukocytes, amplifying platelet activation, and enhancing endothelial dysfunction and proinflammatory actions [2–23]. In addition, polyphosphates released from activated platelets trigger factor V activation and promote factor XI activation via thrombin, which contributes to the synthesis of thicker fibrin strands that are resistant to fibrinolysis [24]. Prophylaxis and treatment of thrombosis can be performed with indirect or direct intervention in these events, such as heparin, inhibitors of coagulation factors, and platelet aggregation inhibitors such as acetylsalicylic acid [25, 26]. COVID-19 is a remarkable thrombotic disease, and the use of prophylactic and therapeutic drugs for treating thrombosis remains controversial, due to the failure to prove its efficacy in clinical trials [27].

Although of great therapeutic value, currently used substances like antiplatelet agents, direct-oral anticoagulants, and low-molecular-weight heparins present serious complications or side effects, the main one being enhanced risk of major bleeding. Therefore, obtaining drugs with the greatest possible antithrombotic effect and the least possible bleeding is still a big issue.

Since (p-BthTX-I)_2_ K displays inhibitory activity against SARS-CoV-2, we sought to investigate its effects on hemostatic parameters and it would be interesting to have a substance with a scope of action that embraced these two mechanisms. (p-BthTX-I)_2_ K prolonged the aPTT and PT time by impairing PKa-like activity from the contact system pathway and FXa-simile activity from the common coagulation pathway. Coagulation factors, which are traditionally considered contributors to the development of venous thrombi are now increasingly implicated in arterial thrombosis. Thus, a therapeutic alteration in aPTT and PT may be important for reducing thrombus formation without increasing the hemorrhagic risk [19].

The relationship between coagulation and inflammation has been noted by studying the physiological response of organisms to different types of proinflammatory stimuli that could activate coagulation flow and phagocytosis of cells through contact. In systemic inflammation, tissue damage can activate the coagulation system, inhibit endogenous anticoagulants, and impair the fibrinolytic response, thereby causing thrombus formation [28]. According to Becker [3], post-mortem examination of COVID-19 patients revealed macro-and microvascular thrombosis in all major organs and mesenteric fat, minimal evidence of microangiopathy, intravascular megakaryocytes, endocardial thrombosis, viral particles in adipocytes, and an excess of platelets in the spleen.

Although several issues regarding the use of antiplatelet and anticoagulant drugs in patients with suspected or confirmed COVID-19 infection remain unclear, antiplatelet agents are recommended due to strong evidence that aggregation inhibition and bleeding control are central therapeutic strategies in combating COVID-19 [29–31].

(p-BthTX-I)_2_ K, as opposed to heparin, inhibited plasma PKa-like activity from 35.5% (5 µg) to approximately 81% (20–80 µg). This may result in decreased kinin production followed by lowered NO release since it may induce arterial dilatation by promoting guanylyl cyclase activation in vascular smooth muscle cells [32]. The therapeutic effects of NO depend on its concentration [33]. NO suppression results in decreased peripheral vasodilation and the consequent cessation of bleeding, as shown by the bleeding time test in mice, a phenomenon common to plasma kallikrein inhibitors [12–35], unlike what happens with heparin, which has a vasodilation-related effect as a consequence of NO release [36].

Despite NO is an inhibitor of platelet activity, (p-BthTX-I)_2_ K has intrinsic antiplatelet activity when stimulated with arachidonic acid, collagen, or ADP, an effect that compensates for the decline in NO concentration and imparts antithrombotic properties to (p-BthTX-I)_2_ K in vivo. Thus, (p-BthTX-I)_2_ K, in addition to demonstrating potent antiviral effects, has shown efficacy in inhibiting arterial thrombosis bleeding in vivo, which makes (p-BthTX-I)_2_ K an excellent candidate for prophylaxis and treatment of COVID-19.

## Data Availability

Datasets used and/or analyzed during the current study are available from the corresponding author upon request.
